# Nutritional Profile and Dietary Patterns of Lebanese Non-Alcoholic Fatty Liver Disease Patients: A Case-Control Study

**DOI:** 10.3390/nu9111245

**Published:** 2017-11-14

**Authors:** Nicole Fakhoury-Sayegh, Hassan Younes, Gessica N. H. A. Heraoui, Raymond Sayegh

**Affiliations:** 1 Department of Nutrition, Faculty of Pharmacy, Saint Joseph University, Damascus Road, P.O. Box 11-5076, Riad el Solh, Beirut, Lebanon; h.gessica10@gmail.com; 2 Department of Nutrition and Health Sciences, Institut Polytechnique UniLaSalle, 19, rue Pierre Waguet, 60026 Beauvais CEDEX, France; hassan.younes@unilasalle.fr; 3 Department of Gastroenterology and Hepatology, Faculty of Medicine, Saint Joseph University, Damascus Road, P.O. Box 11-5076, Riad el Solh, Beirut, Lebanon; rsayegh@usj.edu.lb

**Keywords:** non-alcoholic fatty liver disease, high fruit group, high meat, fast food

## Abstract

Nonalcoholic fatty liver disease (NAFLD) is considered the most common liver disease in the world. Dietary habits have a significant impact on the biological and physical profile of patients and increase the risk of NAFLD. The overall pattern of diet intake is more associated with health outcomes than nutrients. The aim of this study was to evaluate the nutritional profile and the dietary patterns of Lebanese NAFLD patients and compare it with controls. During this study; 112 NAFLD Lebanese adult patients (55 men and 57 women); and 110 controls (44 men and 66 women) were recruited. Dietary intake was evaluated by two 24-h recalls and a semi-quantitative 90-item food frequency questionnaire. Dietary patterns were determined by factor analysis. Results from the study demonstrated that 40% of cases belonged to the high fruit group as compared to 30% following a high meat; fast food dietary pattern. Both groups increased the odds of NAFLD by four-fold (*p* < 0.05). The traditional diet decreases the odds by 33% after adjustment with the covariables. The high fruit diet group was, as with the high meat, fast food dietary pattern, the main potential risk factor for NAFLD in Lebanese patients.

## 1. Introduction

Non-alcoholic fatty liver disease (NAFLD) refers to a spectrum of diseases, ranging from asymptomatic steatosis, to non-alcoholic steatohepatitis (NASH), to cirrhosis [1]. NAFLD is defined as the accumulation of lipids, mainly triacylglycerol, in hepatocytes of individuals who do not consume significant amounts of alcohol (≤2 drinks/day for women, ≤3 drinks/day for men) [2] and in whom other known causes of steatosis, such as certain chronic liver disease (hepatitis A, B, and C, Wilson’s disease) or medications have been excluded. NAFLD is mainly associated with clinical features of metabolic syndrome mainly type 2 diabetes and dyslipidemia [3]. NAFLD patients have more than one feature of the Metabolic Syndrome, and now they are considered the hepatic components of the MS. Several scientific advances in understanding the association between NAFLD and MS have identified insulin resistance (IR) as the key aspect in the pathophysiology of both diseases [4].

Diet composition is an environmental factor that might influence NAFLD severity [5]. Over-nutrition or inappropriate diet, such as high carbohydrate or excessive fat intake, are thought to lead to chronically-elevated glucose, insulin, and free fatty acid concentrations in the blood [6]. Diets with a high glycemic index and glycemic load were found to be positively associated with insulin resistance [7] and the risk of type 2 diabetes, breast cancer, and heart disease [8]. Nutrients, such as monounsaturated fatty acids (MUFAs), are well known to decrease oxidized Low-density lipoprotein -cholesterol (HDL-C) and triacylglycerol concentrations without a decrease in High-density lipoprotein (HDL)-cholesterol [9]. Alanine aminotransferase, triglyceride, serum tumor necrosis factor-alpha levels, as well as fatty liver and fibrosis stage, improved after omega-3, eicosapentaenoic acid (EPA), and docosahexaenoic acid (DHA) supplementation [10,11]. Zinc, ascorbic acid (vitamin C), and α-tocopherol (vitamin E) protect from NAFLD by decreasing intestinal permeability and oxidative stress [12,13].

The consumption of fructose, due to the intake of carbonated-beverages, honey, molasses, candies, and baked food, is increasing significantly. Animal testing has shown fructose-enriched diets to induce the parameters of metabolic syndrome and an increase in the percentage of fat-enriched cells, as well as an increase in fibrosis and in inflammatory markers [14,15].

Studies on the dietary pattern of NAFLD patients are scarce. One prospective study reported a Western-like dietary pattern trend in this population [16] and another one studied the effect of a Mediterranean diet intervention versus a control diet on intrahepatic lipids reduction [17]. The use of dietary pattern in establishing an association between dietary intake and NAFLD is of importance. This will enable the investigation and measure of the overall diet, which is more plausible to be associated with NAFLD than each studied nutrient or single foods [18].

To our knowledge, no previous study was conducted in Lebanon to investigate the dietary patterns that predict NAFLD in Lebanese patients. The primary objective of the present study was to assess the nutritional profile of a sample of NAFLD patients (112 subjects) and compare it with controls (110 subjects). A secondary objective was to compare the dietary patterns between the two groups.

We hypothesized that there is a strong association of the high meat, fast food (Western-like dietary pattern) with NAFLD patients and an inverse association between traditional dietary pattern and the disease.

## 2. Materials and Methods

### 2.1. Study Design

From November 2013 until June 2016, 528 Lebanese patients seen consecutively in an outpatient clinic of the department of gastroenterology in an academic hospital in Beirut were invited to participate in the study. These patients were coming for a routine health check. At enrollment, they provided their informed consent to participate in the study. Of these 528 patients, 400 eligible Lebanese patients were newly diagnosed as having NAFLD (identified as cases), and the remaining 128 patients were free of liver disease and, hence, served as controls.

However, only 320 Lebanese patients with complete clinical, anthropometric, and dietary data were enrolled, of whom 112 NAFLD cases were selected to age-gender match 110 controls who also met the same criteria. The sample size for this study (*n* = 222) was statistically significant using an alpha of 0.05 and a power of 90% [19] considering a significant outcome measure (odds ratio (OR)) of 1.45 [20].

### 2.2. Eligibility Criteria

The study sample included consecutive visits of patients coming to this outpatient clinic. Eligible cases for this study were men and women aged between 18 and 70 years old. Patients were included in the study if they were recognized as Lebanese and without: (1) biliary diseases or recognized cirrhosis; (2) infection with hepatitis A, B, or C virus; (3) genetic metabolic disease; (4) auto-immune liver diseases; and (5) diabetes type 1. Additional inclusion criteria were: (1) non-pregnancy among women; (2) less or equal to 2 servings/day of alcohol consumption; and (3) absence of drugs inducing hepatotoxicity (tamoxifen, steroids, amiodarone). The controls were selected according to the same criteria.

### 2.3. Study Protocol

**Figure 1:**
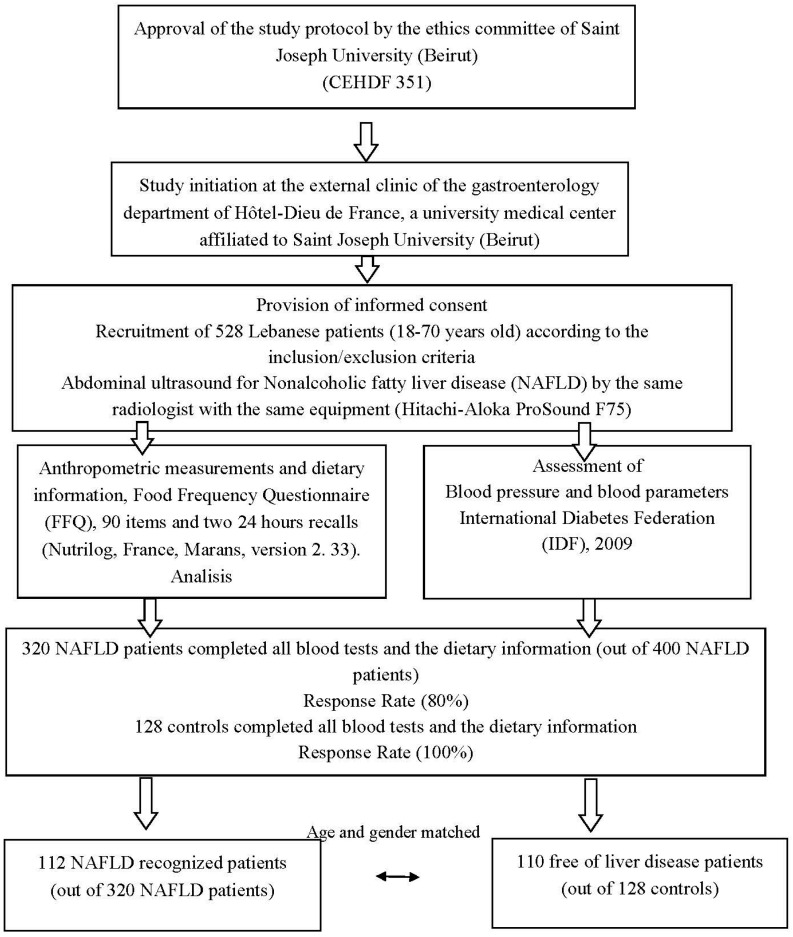
Flowchart for selection and enrollment of study subjects (cases and controls). NAFLD: Nonalcoholic fatty liver disease; FFQ: Food Frequency Questionnaire.

Following the provision of informed consent, all participants underwent an abdominal ultrasound by the same radiologist with the same equipment (Hitachi-Aloka ProSound F75, Tokyo, Japan) and were classified in the two groups accordingly. The recruitment and selection of the eligible Lebanese patients are represented in the flowchart (Figure 1). The liver steatosis was estimated with the evaluation of image brightness of the echo pattern. Abdominal ultrasound cannot identify hepatic fat deposition if it is less than 33% of the total liver weight and, accordingly, all patients with a lower percentage were categorized as controls. Laboratory data were collected from fasting NAFLD patients (more than 12 h of fasting) and controls at enrollment after informed consent. Anthropometric measurements and dietary information were obtained using a questionnaire which included a food frequency questionnaire (FFQ) [21] adapted to typical Lebanese diet and two 24-h recalls (typical week and week-end days that characterize the patient diet (Supplementary Materials).

To avoid reporting bias, patients were interviewed either prior to the performance of the abdominal ultrasound or prior to their knowledge of the abdominal ultrasound results. The questionnaire contained duplicate questions to minimize social and memory bias. The questionnaire was administered through face-to-face interview and carried out on all patients by the same nutritionist. For the two 24-h recalls used, participants were asked to recall their intake on a typical week day and a weekend day during the same interview.

### 2.4. Validity and Reproducibility of the Test

A pre-test (two 24-h recalls and the FFQ questionnaire) was administered to 50 patients at the start of the study, prior to diagnosis, to evaluate the reproducibility of the questionnaire. The re-interview of this sub-sample by the same nutritionist after one month yielded an interclass correlation coefficient (ICC) = 0.957 (0.917–0.978), *p =* 0.0001 for energy intake/day of all participants. According to macronutrient intake/day, such as the percentage of carbohydrates and proteins of total energy intake/day, ICC varies between 0.969 (0.939–0.984) and 0.961 (0.924–0.980), respectively (*p =* 0.0001). This coefficient corresponds to the agreement in energy intake/day (kcal/day) and in macronutrients intake/day (g) or in their percentage of the total energy intake at two times point for each participant. The estimate of validity was performed, using Bland-Altman analyses, on 100 patients, prior to diagnosis, who fulfilled both the FFQ questionnaire and the two 24-h recalls [22]. Results for the percentage of energy from carbohydrates, fat, and proteins are shown, respectively, in Figures S1–S3 (Supplementary Materials). The difference in dietary intake between the FFQ and the mean of estimated nutrients of both 24-h recalls was plotted on the Y axis and the mean intake of both tools on the X axis. Most data points were clustered around the mean difference line between the two limits of agreement.

### 2.5. Anthropometric Data

All anthropometric data were carried out by the same nutritionist. Body weight and height were measured to the nearest 0.1 kg and 0.1 cm, respectively, using an upright scale (Seca, 402 KL, PH, Hamburg, Germany). Body mass index (BMI), was calculated as weight divided by height squared (expressed as kg/m^2^). Patients were categorized as obese, overweight, or normal weight according to the World Health Organization (WHO) obesity classification of 2004: normal weight (BMI between 18 and 24.9 kg/m^2^), overweight (BMI between 25 and 29.9 kg/m^2^), and obese (BMI ≥ 30 kg/m^2^). The waist circumference (cm) (cut-off values >80 cm for women, >94 cm for men) [23], and the waist/hip ratio (cut-off values >0.85 for women, >0.90 for men) were also assessed in both groups (WHO, 2008). The waist circumference (measured at the midpoint between the lowest rib and the iliac crest) and the waist to hip ratio (the hip was measured as the greatest part of the buttock) was measured to the nearest 0.1 cm using a tape measure. All physical measurements were measured three times and the average of the three measurements was recorded.

### 2.6. Clinical Data of Cases

All parameters corresponding to the criteria of the metabolic syndrome [17] were evaluated in both groups as follows: systolic blood pressure ≥130 mmHg, diastolic blood pressure ≥85 mmHg, fasting serum glucose ≥5.5 mmol/L, fasting serum triglycerides ≥1.7 mmol/L, fasting serum HDL < 1.0 mmol/L for males, and <1.3 mmol/L for females. All biochemical assessments were performed at the hospital laboratory by standard laboratory methods. Blood pressure (systolic and diastolic) was taken using a mercury sphygmomanometer. Patients who were on drugs for hypertension, hyperglycemia, or dyslipidemia were classified with metabolic syndrome regardless of laboratory findings. The homeostasis model assessment of insulin resistance (HOMA-IR), an index of insulin resistance (IR), was calculated for both groups as fasting serum insulin (µIU/mL) × fasting serum glucose (mmol/L)/22.5. The values which were higher than the value of 3 indicated a state of IR [24,25].

### 2.7. Food Consumption Patterns

Food consumption patterns were evaluated using professional nutrition software (Nutrilog, Marans, France, version 2.33) to calculate and record daily intakes. The questionnaire used consisted of two parts. The first part contained questions relative to food consumption patterns, such as frequency of meals/day, frequency of consumption of vegetables/day, and the kind of dairy products or meat consumed, generally. Open questions were also asked such as “What kind of oil do you use for frying, baking or for salad dressings?” The questionnaire also contained continuous variables, such as age (years), crowding index (total number of co-residents per household, excluding newborn infants, divided by the total number of rooms, excluding the kitchen and the bathrooms) and BMI (kg/m^2^) and categorical variables, such as gender, marital status, and occupation. The second part was a FFQ of 90 items [21] adapted to Lebanese food and translated into Arabic. Questions were on consumption frequency of fruits, dairy products (whole, semi-skimmed or skimmed), cereals, and vegetables (daily, weekly, or monthly), consumption frequency of items rich in simple carbohydrates, such as carbonated beverages, chocolate, or honey (daily, weekly, or monthly), and the frequency of meat, fish, seafood, and chicken eaten (daily, weekly, and monthly). The selected frequency category for each food item (monthly, weekly, or daily) was converted to a daily intake in grams. Total nutrient intake was calculated from the sum of the frequency weight products and nutrient content of the portion of food; once a day = 1, four times a week = 4/7, once in a month = 1/30 (dietary information and the FFQ are present in the Supplementary Materials).

The two 24-h dietary recalls were used to add any items (such as cheese, milk, or others) that had not been mentioned in the FFQ and to determine the amount and the intake frequency/month, week/day, since participants usually memorize what they eat on a typical week or a week-end day. A typical week day and a weekend day were chosen since people modify their eating patterns at the end of the week. The participant had to recall all food items and drinks with all the details. The two 24 h recalls were also used to report the local and traditional dishes usually eaten at lunch or as a dinner. For complex recipe, nutrients values were obtained from the standardized recipes derived from the databases of the American University of Beirut. For any lacking one, the recipe was cooked, ingredients split to a manual analysis, and added to the software as extra recipes [26]. The software analyzed daily energy intake, macronutrients (carbohydrates, proteins, fat), cholesterol, simple carbohydrates, fiber, micronutrients (zinc), and vitamins C and E. The patients were asked to describe the portion size of each food by comparing with food photographs (Numed s.a.r.l, Beirut, Lebanon). This method was found to be of benefit in estimating food-portion size and the nutrient content of the meal [27].

The American University of Beirut database of 1970 was used for some national ingredients or recipes that were not found in the United States Department of Agriculture (USDA) [28]. Dietary fructose was calculated by the sum of natural fructose (NF) (free fructose level and/or fructose coming from sucrose) present in fruits, honey, molasses, and beverages or added sugar, such as those present in commercial foods. Dietary simple carbohydrate was defined as the total intake of disaccharides and monosaccharides naturally present in food and the added sugar from commercial foods

While determining dietary patterns, food items were grouped into 25 groups according to food family and nutrient profile (Supplementary Materials). The total consumption for each food group was determined by summing the daily intake of servings from each item in this group. The correlation matrix table was examined between items and checked statistically to consider the use of factor analysis. The Kaiser Meyer-Olkin measure of sampling adequacy value was 0.768. The Bartlett’s test of sphericity value was significant (*p* < 0.0001). The number of components to extract was based on the Kaiser criterion (eigenvalues > 1), the change in the shape of the scree plot, and the loading of the items in the components generated (component matrix). Varimax rotation was conducted and dietary patterns were named according to food groups with a factor loading greater than 0.3. Each participant had a factor score for each dietary pattern. The factor score for each participant was calculated by the Anderson and Rubin (1956) method [29].

### 2.8. Demographic and Lifestyle Data

Demographic data, such as age, gender, occupation, education, and marital status were recorded. Questions considering current smoking, presence of physical activity, and kind and duration of individual activity were administered. The participant was considered as a current smoker if he smokes ≥1 cigarettes/day, 1 cigar/day, or 1 water pipe/week [30]. The type and duration of physical activity, including habitual work and leisure physical activity in both groups were assessed by referring to Center of Disease Control (CDC) guidelines of 1996. Physical activity was defined as either moderate (3.5–7 kcal/min), such as moderate housework, walking, gardening, and recreational swimming, or vigorous (>7 kcal/min), such as tennis, football, and non-recreational swimming [31]. Patients were considered active if their vigorous activity surpassed 20 min/day and was undertaken continuously at least 3 times/week, or their moderate activity surpassed 30 min/day for at least 5 times/week and started at least three months prior the study [32].

Type 2 diabetes, hypertension, and cardiovascular diseases (CVD) were considered present if the patient had been diagnosed of having any of these conditions or if they were taking medications for these diseases. Family history of obesity, type 2 diabetes, hypertension, dyslipidemia, and CVD were recorded and restricted to first degree relatives (parents, brothers or sisters).

### 2.9. Ethical Considerations

The procedures followed in the study were in accordance with the revised form of Helsinki declaration, 1975. The study protocol was approved by the university ethics committee (CEHDF 351) and the study was supported with grants by the research council of Saint Joseph University, Lebanon. All patients provided written informed consent upon enrollment. All data were kept confidential and the anonymity of respondents was maintained.

### 2.10. Statistical Analysis

Continuous variables were expressed as means and standard deviation. Geometric means (Log10 of quantitative variables) were used in the case of the absence of normal distribution. For comparison between continuous data, the independent samples *t*-test was used. The association between proportions or percentage was tested using χ^2^-test. Spearman’s correlation coefficients were used to determine the correlation between anthropometric and food parameters and dietary patterns. Energy adjustment was carried out using the regression residual method [32]. Multivariate logistic regression was conducted to correlate dietary patterns with the absence or presence of NAFLD. The score of each dietary pattern was entered as an independent variable with other co-variables. All tests were two-tailed and the significance level was set at *p* < 0.05. The statistical analysis was carried out using SPSS 20 for Windows (IBM Corp., Released 2011, IBM SPSS Statistics for Windows, Version 20.0. Armonk, NY, USA).

## 3. Results

### 3.1. Patients’ Characteristics

A total of 112 cases were included in the study. The mean age for cases was 39.9 ± 6.0 years (49.1% men versus 50.9% women). In comparison, the mean age for controls (40% men versus 60% women) was 38.8 ± 13.2 years (Table 1). Participants were residents from all of Lebanon’s six governorates and formed an approximate representation of the national population. In total, 54.5% of our cases had a university degree, as compared to 86.3% for controls. Eighty percent of cases and 56.4% of controls were married (Table 1). Seventy-seven percent of cases and 69.1% of controls were of high or middle socioeconomic status. Socioeconomic status was calculated by the crowding index. No significant differences in socioeconomic status, denoted by the crowding index, as well as the place of birth, were reported between cases and controls (*p* > 0.05), yet, significant differences according to place of residence, marital status, academic level, and occupation were found between cases and controls (*p* < 0.05).

A total of 62.5% of cases had three parameters or more for metabolic syndrome as compared to 12.7% for controls and 18.8% of cases were type 2 diabetes versus 0.9% of the controls (Table 2). Seven percent of controls and 49.1% of cases had elevated serum triglycerides, while 84.8% of cases versus 77.3% of controls had elevated fasting blood sugar. A significant difference in family history with type 2 diabetes, obesity, liver, and CVD was detected between the two groups (*p* = 0.0001) (Table 2). While comparing the clinical characteristics between the two groups, 25% and 26% of cases had systolic or diastolic hypertension and 0.9% had cardiovascular diseases (Table 2). In comparison, 6.4% and 3.6% of controls were systolic and diastolic hypertensive and none had cardiovascular diseases.

Regarding physical activity, 18.8% of cases participated in some type of physical activity of whom 13.4% were moderately active and 5.4% were vigorously active. In comparison, 50.9% of controls were active (24.5%; moderate activity and 26.4% were vigorously active) (Table 2).

### 3.2. Anthropometric Parameters

A total of 55.3% of cases were obese (BMI ≥ 30 kg/m^2^) as compared to 8.2% for controls (Table 2). The percentage of cases having a raised waist circumference (≥94 cm) was 85.7% for males and 82.2% for females (waist circumference ≥ 80 cm) (*p* = 0.0001), (Table 2). According to waist/hip ratio, no significant difference was present in male cases as compared to male controls. In contrast, the percentage of female cases was significantly different from female controls (*p* = 0.0001), (Table 2).

### 3.3. Dietary Data

Table 3 shows the comparison of dietary data between controls and cases. The mean values of energy intake/day (BMI < 30 kg/m^2^), (physical activity no/yes), simple carbohydrate, fructose, and fructose coming from fruits were significant different between cases and controls.

### 3.4. Dietary Patterns and Their Correlates with Energy and Macronutrient Intakes

**Figure 2:**
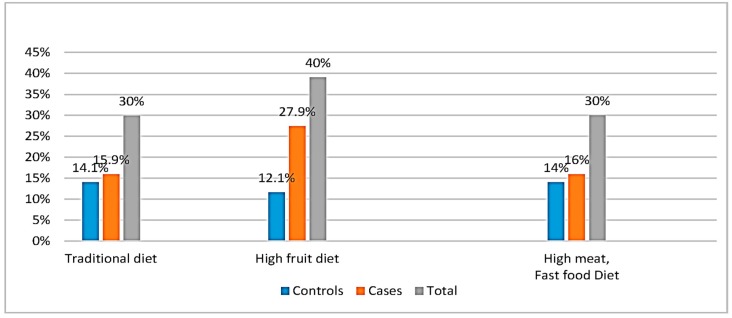
Distribution of participants according to the different dietary patterns.

**Figure 3:**
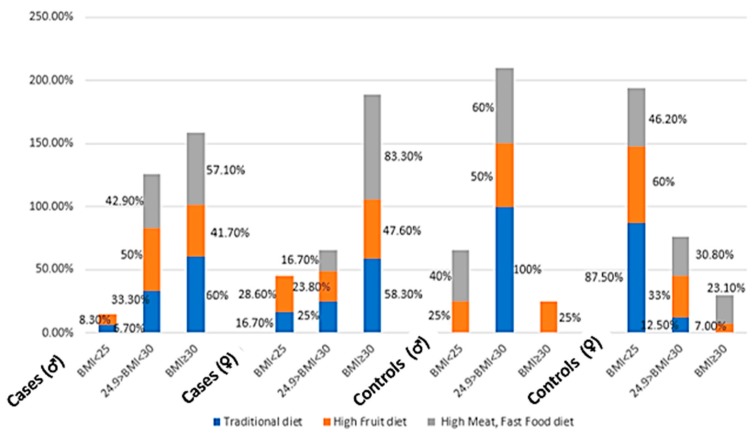
Distribution of participants according to the different dietary patterns, BMI, and gender.

Three dietary patterns were generated by the factor analysis explaining in total 32.70% of the total variance (Table 4). Thirty percent of participants followed the traditional diet (15.9% cases, 14.1% controls) as compared to 40% and 30% adhering to the high fruits (27.9% cases, 12% controls) and the high meat, fast food diet (16% cases, 14% controls), respectively (Figure 2). Figure 3 showed the distribution of participants according to the three dietary patterns, BMI and gender. A high percentage of female, obese cases and controls (BMI ≥ 30 kg/m^2^) followed a High meat, Fast Food diet while for men, the obese controls followed exclusively a High Fruit diet. The first model generated (15.53% of the total variance) was the traditional one which consisted of items such as legumes; chickpeas, corn and peas; or vegetables, such as cauliflower, pepper, and lettuce. The second model was the high fruits model explaining 9.74% of the total variance and the last model (high meat, fast food) accounts for 7.43% of the total variance. Table 5 shows the significant association demonstrated by Spearman’s correlation between the factor scores of the high meat, fast food diet and the high fruits dietary pattern with sucrose, fructose, and energy intake (*p* < 0.01). The table also demonstrated the significant correlation between fat, saturated fatty acid, and carbohydrate intake with the high meat, fast food dietary pattern (*p* < 0.01).

Table 7 shows the dietary distribution of different food groups between cases and controls. Results were adjusted for patients (cases and controls) consuming more than 3000 kcal/day for men and more than 2000 kcal/day for women.

## 4. Discussion

This study confirmed the high prevalence of metabolic syndrome (62.5%) in cases with significant difference in its parameters as compared to controls (12.7%) (Table 2). Marchesini et al., considered NAFLD as an additional feature of the metabolic syndrome [37]. The latter is usually associated with central obesity, dyslipidemia, and type 2 diabetes [38]. In our study, 55.3% of our cases were obese (BMI ≥ 30 kg/m^2^) as compared to 8.2% for controls, (*p =* 0.0001). The percentage of cases (males, females) having a waist circumference (cm) higher than the International Diabetes Federation (IDF) 2009 criteria was significantly higher in NAFLD cases in comparison with controls (*p* = 0.02, *p* = 0.001). These are typical findings for NAFLD patients, knowing that an increase in the waist circumference (cm) or in the waist/hip ratio indicates an accumulation of intra-abdominal adipose tissue responsible for the development of metabolic syndrome and liver steatosis [38]. Furthermore, 18.8% of cases were associated with type 2 diabetes, 0.9% with cardiovascular diseases, and nearly 50.9% with hypertension (mmHg) (Table 2). In the control group, 10% were hypertensive and none had CVD with almost an absence of type 2 diabetes. This is not a new finding knowing that type 2 diabetes and NAFLD are particularly closely related. The latter is associated with dysfunctional adipose tissue and associated with insulin resistance and pancreatic beta cell dysfunction [39]. In addition, family history of cardiovascular disorders, diabetes, and hypertension was more prevalent in cases than in controls. Chehreh et al., reported positive family history for CVD, diabetes, dyslipidemia and hypertension among patients with NAFLD suggesting that the disease is multifactorial, involving either hereditary or environmental factors with possible interaction [40].

Elevated HOMA-IR and serum triglycerides were characteristics of NAFLD patients (Table 2). This may be explained by the increase in triglycerides and free fatty acids released from adipose tissues, mainly visceral ones. These interfere with insulin signaling by modulating insulin receptor substrate-2 (IRS-2) phosphorylation [2]. Insulin resistance status is generally accompanied by impaired serum clearance of Very-low-density lipoprotein (VLDL) and intestinally-derived chylomicrons which result in elevated serum triglycerides [41].

Regarding environmental factors, significant differences in physical activity and smoking were found between the two groups (Table 2). Smoking is well known to be a source of oxidative stress, while exercise is known to reduce metabolic syndrome [42]. A large-scale study by Church et al., (*n* = 218 men) reported an inverse association between cardio respiratory fitness and the prevalence of NAFLD [43]. Hannukainen et al. and Tamura et al. demonstrated that hepatic triglycerides accumulation decreases with exercise and increase delivery of glucose and insulin to the muscles [44,45].

Several studies reported an association between food patterns and metabolic diseases, such as T2D [46] and obesity or cardiovascular diseases [47]. Few reported on NAFLD [16,48]. According to food dietary patterns between the two groups, the results obtained were in line with other studies [16,48]. However, our study generated a new dietary pattern, the high fruit group. This confirmed the fact that fruits are an important part of the Lebanese diet and constituted a whole independent entity.

Twenty-eight percent of cases belonged to this group as compared to 12.1% of controls (Figure 2). The high consumption of fruits was significantly different between cases and controls (women) after adjustment for energy intake/day, (*p* < 0.05) (Table 7). The fruits consumed were mainly plums, raisins, apples, and figs. These kinds of fruits are highly available on the Lebanese market for a relatively low cost, besides being a characteristic of the traditional diet. Other studies have found this high consumption of fruits, comparable to the results obtained, in other Mediterranean populations [49,50]. Fruits are well known to be rich in free fructose levels, as well as in simple carbohydrates. This result was confirmed by the significant correlation between fructose and sucrose intake/day and scores of the high fruit group pattern (*r* = 0.307, *r* = 0.252), respectively, (*p* < 0.05). Molasses and honey are also rich in these monosaccharides and disaccharides. Honey is usually used as a replacement for sugar in most hot beverages, mainly in tea, while molasses is mainly consumed in desserts. Nevertheless, these two items showed small loading in the three components (less than 0.3). Carbonated beverages enriched in these monosaccharides and disaccharides loaded very low in the fruit group (<0.3), but loaded high in the fast food group (>0.3). The novelty in our study is that the high fruit group pattern increased the odds of NAFLD by four-fold, *p* < 0.05, after adjustment with its covariables, and the mechanism behind this may be through the provision of large amounts of sugars, such as fructose [51]. The consumption of fructose has been linked to NAFLD, as well as various aspects of the metabolic syndrome, including dyslipidemia, visceral adiposity, insulin resistance, and high blood pressure [52].

Meat such as pork, chicken, beef meat and hotdog loaded high in the high meat, fast food group. Both groups consumed largely beef, chicken, or lamb meat (Table 7) with almost the same percentage adhering to the high meat, fast food group. The significant difference in energy intake (kcal/day) and the frequency of red meat consumed between cases and controls could justify the fact that the control group is NAFLD-free. A study done by Zelber Sagi et al. [20] showed that all types of meat were significantly associated with an increased risk for NAFLD. Other items, such as pizza, fries, cream, ketchup, and other condiments also loaded high in this diet group, confirmed by the significant correlation between sucrose intake/day and scores of this diet group. This kind of diet characterized by high intake of pasta, red meat, desserts, and pizzas triggered an increase in weight, a higher postprandial insulin secretion and, ultimately, an insulin resistance. This will increase liver fat storage via the de novo lipogenesis pathway [16].

Food, such as fish, sardine, salmon, and tuna, loaded low (factor loading matrix < 0.2) in both the traditional and the high meat, fast food pattern groups. These items were rarely eaten among the Lebanese populations. The average consumption of fish and other seafood was well below the recommended servings/week (Table 7). Although Lebanon is a coastal country, the Lebanese population avoids eating seafood for cultural, economic, and public health reasons. The general belief is that the coastline is polluted, and consumable fish are unavailable or very expensive. A study done by Nasreddine et al., reported this low consumption of seafood in Lebanese subjects with 73.6% of Lebanese adult participants consuming less than two servings of fish per week [53].

Milk, cheese, labneh (a local soft creamy cheese), coffee, and tea also loaded weakly in the three groups. This can be explained by the fact that these items are equally consumed by participants, except for milk, which is considered as a flatulent item, and is consequently avoided by the general population. Vegetable oils and olives loaded equally in the first two components (traditional and high fruits groups). This reflects the main feature of the Lebanese traditional diet which is enriched in oil, mainly olive oil.

Our three groups’ results confirmed the statement that the Lebanese traditional dietary pattern is still present in a subgroup of our population and is highly representative of the Mediterranean diet, composed mainly of vegetables, seeds, olive oil, and legumes. However, the presence of the high meat, fast food group reveals a trend towards an animal-based westernized food pattern rich in proteins and refined cereals. The presence of the high fruit dietary pattern group is in line with the dietary culture trend and tradition of the people living around the Mediterranean basin.

Some limitations and bias were present in the study. The first limitation was the difficulty in matching BMI (kg/m^2^) between controls and cases. For a period of two and a half years, only 8.2% of controls were classified as obese (BMI ≥ 30 kg/m^2^). The second limitation was the ultrasound used to assess the presence or not of fatty liver. The limitation is its low sensitivity for mild steatosis (<33%) and to accurately quantify fatty infiltration [54]. However, this technique still represents the first-line diagnostic tool for simple liver steatosis. It had been widely used in different studies, since it is a non-invasive method with a sensitivity of 60–94% and a specificity of 66–95% [48,55]. The third limitation stems from recall bias among patients. They may over-report physical activities, as well as under-report dietary intake, provide incorrect estimations of portion sizes, or have memory loss. To overcome these limitations, patients were interviewed and reported their dietary intake prior the disease diagnosis or prior to any diet change due to medical advice or drugs used. Another limitation came from the use of factor analysis which requires subjective decisions for grouping food for analysis or in choosing the method of rotation or for determining dietary patterns according to their loading factors.

## 5. Conclusions

This study sheds light on a new dietary pattern group, the high fruit pattern. A diet which contains more than 2–3 servings/day of fruits (>20 g/day of fructose) could be an added source of fructose and sucrose besides the often-cited carbonated beverages and commercial fructose enriched food and pastries. This study encourages the consumption of a traditional dietary pattern. This type of diet, associated with weekly exercise and a total energy intake/day close to the daily recommended intake (DRI) may be beneficial to NAFLD patients. This may constitute a first-line therapy before a possible progression from simple steatosis to steatohepatitis and cirrhosis.

## Figures and Tables

**Table 1:** Comparison of age and sociodemographic data between cases and controls.

	Cases (*n* = 112)	Controls (*n* = 110)	*p*-Value	Total (*n* = 222)
Age (years), mean ± SD	39.9 ± 6.0	38.8 ± 13.2	0.39	
Gender				
Men, *n* (%)	55 (49.1)	44 (40)	0.17	99 (44.6)
Women, *n* (%)	57 (50.9)	66 (60)		123 (55.4)
Place of birth, *n* (%)				
Mount Lebanon	17 (15.2)	29 (26.4)	0.09	46 (20.7)
North	15 (13.4)	10 (9.1)		25 (11.3)
South	15 (13.4)	14 (12.7)		29 (13.1)
Beirut	42 (37.5)	39 (35.5)		81 (36.5)
Bekaa	16 (14.3)	13 (11.8)		29 (13.1)
Nabatieh	5 (4.5)	-		5 (2.3)
Abroad	2 (1.7)	5 (4.5)		7 (3.2)
Place of residence, *n* (%)				
Mount Lebanon	37 (33.0)	56 (50.9)	0.0001	93 (41.9)
North	12 (10.7)	4 (3.6)		16 (7.2)
South	7 (6.3)	2 (1.8)		9 (4.0)
Beirut	30 (26.8)	38 (34.5)		68 (30.6)
Bekaa	12 (10.7)	7 (6.4)		19 (8.5)
Nabatieh	10 (8.9)	-		10 (4.5)
Abroad	4 (3.6)	3 (2.7)		7 (3.2)
Marital Status, *n* (%)				
Unmarried	18 (16.1)	46 (41.8)	0.0001	64 (28.8)
Married	90 (80.4)	62 (56.4)		152 (68.5)
Divorced	3 (2.7)	2 (1.8)		5 (2.3)
Widow/er	1 (0.9)	-		1 (0.5)
Academic level, *n* (%)				
Illiterate	-	-	0.001	-
Elementary	9 (8)	1 (0.9)		10 (4.5)
Intermediate, secondary	42 (37.5)	14 (12. 8)		56 (25.2)
University	61 (54.5)	95 (86.3)		156 (70.2)
Occupation, *n* (%)				
Self-employed	39 (34.8)	33 (30.3)	0.0001	72 (32.4)
Employee	41 (36.6)	53 (48.2)		94 (42.3)
Retired/unemployment	-/26 (23.2)	7/3 (6.4/2.8)		7/29 (3/13.1)
Others	6 (5.4)	14 (12.7)		20 (9.0)
Crowding index ^†^, *n* (%)				
≤1	86 (76.8)	76 (69.1)	0.88	162 (73.0)
>1	31 (27.7)	29 (26.4)		60 (27.0)

Continuous variables are reported as geometric means ± standard deviations (SD); Statistical test used: independent *t* test; Categorical variables are reported as numbers and percentage; Statistical test used: χ^2^-test; ^†^ Crowding index: number of co-residents by room.

**Table 2:** Comparison of clinical and environmental data between controls and cases.

	Cases (*n* = 112)	Controls (*n* = 110)	*p-*Value
Presence of metabolic syndrome, *n* (%)	70 (62.5%)	14 (12.7%)	0.0001
Hypertension ≥ 130/85 (mmHg), *n* (%) ^§^	28 (25%)/29 (25.9%)	7 (6.4%)/4 (3.6%)	0.041/0.002
Fasting blood sugar ≥ 5.5 mmol/L, *n* (%) ^§^	95 (84.8%)	85 (77.3%)	0.072
Triglycerides ≥ 1.7 mmol/L, *n* (%) ^§^	55 (49.1%)	8 (7.3%)	0.0001
HDL-C < 1 mmol/L (M), *n* (%) ^§^	14 (12.5%)	2 (1.8%)	0.031
<1.3 mmol/L (F), *n* (%) ^§^	61 (54.5%)	27 (24.5%)	0.003
Waist circumference (cm) (M) ≥ 94 ^§^	96 (85.7%)	63 (57.3%)	0.002
(F) ≥ 80 ^§^	92 (82.2%)	40 (36.4%)	0.0001
Waist/hip ratio (M) > 0.90 ^§^	106 (94.6%)	91 (82.7%)	0.06
(F) > 0.85 ^§^	90 (80.4%)	56 (50.9%)	0.0001
Obesity (yes), *n* (%)	62 (55.3%)	9 (8.2%)	0.0001
Diabetes type 2 (yes), *n* (%)	21 (18.8%)	1 (0.9%)	0.0001
HOMA-IR > 3, *n* (%)	53 (47.3%)	12 (10.9%)	0.0001
Cardiovascular disease (yes), *n* (%)	1 (0.9%)	0 (0%)	0.321
Family medical history (yes), *n* (%)	97 (86.6%)	78 (70.9%)	0.004
Smoking (yes), *n* (%)	47 (41.9%)	29 (26.4%)	0.016
Physical activity, (yes) *n* (%)	21 (18.8%)	56 (50.9%)	0.0001
Moderate, *n* (%)	15 (13.4%)	27 (24.5%)	0.03
Vigorous, *n* (%)	6 (5.4%)	29 (26.4%)	0.008

Test χ^2^-test (categorical variables); ^§^ Reference values correspond to the criteria of IDF, 2009 for clinical diagnosis of the Metabolic Syndrome; Abbreviations: BMI, Body mass index; HDL-C, High density lipoprotein-Cholesterol; HOMA-IR, Homeostasis model assessment of insulin resistance; M, Male; F, Female.

**Table 3:** Comparison of dietary data between controls and cases.

	Cases (*n* = 112)	Controls (*n* = 110)	*p-*Value
Energy intake, (kcal/day), mean ± SD (BMI < 30 kg/m^2^)	3548.13 ± 0.20	2238.7 ± 0.17	0.0001
Energy intake) (kcal/day), mean ± SD (BMI ≥ 30 kg/m^2^)	3835.3 ± 0.16	3784.4 ± 0.22	0.935
Energy intake, mean ± SD (Physical activity, no)	3775.72 ± 0.19	2383.96 ± 0.22	0.0001
Energy intake (kcal/day), mean ± SD (Physical activity, yes)	3348.11 ± 0.15	2301.44 ± 0.15	0.0001
% of energy from carbohydrate, mean ± SD	45.7 ± 0.09	43.7 ± 0.08	0.124
% of energy from protein, mean ± SD	13.8 ± 0.14	14.5 ± 0.11	0.446
% of energy from fat, mean ± SD	37.2 ± 0.10	38.9 ± 0.07	0.099
Simple carbohydrate (g/day), mean ± SD	131.8 ± 0.24	89.12 ± 0.23	0.0001
Fructose (g/day), mean ± SD	57.5 ± 0.29	38.0 ± 0.22	0.0001
Fructose (g/day) coming from fruits, mean ± SD	22.4 ± 0.34	15.1 ± 0.33	0.0001

Continuous variables are reported as geometric means ± standard deviations. Statistical test used: independent *t*-test; SD, standard deviation. Energy intake/day had been adjusted for BMI (</≥30 kg/m^2^) and for physical activity (no/yes) Energy intake/day, % of energy from carbohydrate, % of energy from proteins and from fat, simple carbohydrate (g/day), fructose, and fructose present in fruits (g/day) had been analyzed using the United States department of agriculture (USDA) [27], (Nutrilog, Marans, France, version 2.33).

**Table 4:** Factor loading matrix for the three identified dietary patterns in the study population.

Food Group	Patterns		
	Traditional Lebanese	High Fruits	High Meat, Fast Food
Vegetables	0.69		
Chickpeas, red beans, lentils, peas	0.44		
Fruits and fruit juices		0.71	
Vegetable oil/olives	0.28	0.29	
Fish and sea food		0.35	
Almonds, walnuts, hazelnuts, sesames		0.20	
Desserts, Arabic pastries			0.23
Beef meat			0.59
Hamburger			0.58
Fries			0.56
Pork			0.56
Pizza			0.53
Spaghetti or noodles or cooked rice			0.48
Chicken			0.49
Carbonated beverages			0.46
Pies or fatayer			0.45
1 chicken egg			0.44
Fresh cream			0.42
Mayonnaise or mustard			0.41
Ketchup			0.41
Chips			0.36
Hot dog			0.35
Energy drink			0.28
Ham			0.28
Milk chocolate			0.21
Percent variance explained by each pattern	15.53%	9.74%	7.43%

Extraction method: principal component analysis; Rotation method: Varimax with Kaiser normalization; Absolute values <0.2 were excluded from the table.

**Table 5:** Spearman’s correlation coefficient between pattern scores and anthropometric measurements, nutrients, and total energy intake.

	Traditional Lebanese	High Fruit	High Meat Fast food
BMI (kg/m^2^)	0.188 *	0.148	0.183 *
Energy intake/day (kcal)	0.122	0.284 **	0.468 **
Carbohydrates (g)	0.180 *	0.154	0.377 **
Protein (g)	0.214 **	0.233 **	0.391 **
Fat (g)	0.156	0.162 *	0.437 **
Saturated fatty acids (%)	0.113	0.032	0.363 **
Cholesterol (g)	0.039	−0.037	0.337 **
Polyunsaturated fatty acid (%)	−0.181 *	−0.023	0.111
Sucrose (g)	0.153	0.252 **	0.234 **
Fructose (g)	0.149	0.307 **	0.240 **
Dietary fiber (g)	0.222 **	0.235 **	0.039
Omega-3 (g)	0.195 *	0.211 **	0.133
Omega-6 (g)	0.182 *	0.081	0.177 *

Adjustment for energy was done by residual method described by Willet [33]. * Correlation is significant at *p* < 0.05; ** Correlation is significant at *p* < 0.01. The association between the dietary patterns and the odds of NAFLD was highlighted in Table 6. Both high meat, fast food and high fruit dietary pattern groups were associated with a four-fold increase in the odds of NAFLD (OR: 4.081, CI: 1.356–12.28) (OR: 4.061, CI: 1.320–12.10), respectively, after adjustment with co-variables (Model 2), while scores of the traditional pattern were associated with low odds of NAFLD (OR: 0.303, CI: 0.107–0.859).

**Table 6:** Odds ratios and 95% confidence intervals for the association between dietary patterns and the odds of NAFLD in the study population.

	Traditional Lebanese		High Fruit Group		High Meat, Fast Food Group	
	OR	CI	OR	CI	OR	CI
Model 1 *	0.349	0.135–0.906	3.092	1.151–8.049	3.192	1.251–8.149
Model 2 **	0.303	0.107–0.859	4.061	1.320–12.10	4.081	1.356–12.28

* Model 1 is adjusted for presence of metabolic syndrome (yes/no), energy intake (Kcal/day) and education (illiterate, primary, high school, and university); ** Model 2 is adjusted for variables in Model 1, as well as physical activity (no/yes), family history (yes/no), smoking (yes, no), place of residence and profession (freelance, employee, unemployed, retirement, and others). OR, Odds Ratio; CI, Confidence Interval.

**Table 7:** Dietary patterns of cases and controls.

(*n* = 222)	Cases (*n* = 112)	Controls (*n* = 110)	*p*-Value
Red or organ meat consumed, ≥5–6 servings/week or more	(M): 20 (43.5%)	(M): 9 (69.2%)	(M): 0.101
	(F): 29 (63.5%)	(F): 9 (28.1%)	(F): 0.002 *
Raw or cooked vegetables, less than 2 servings/day	(M): 35 (74.5)	(M): 4 (30.8)	(M): 0.003 *
	(F): 30 (65.2%)	(F): 15 (46.9%)	(F): 0.107
Fruits consumed, 3–5 servings/day or more	(M): 24 (51.5%)	(M):10 (71.4%)	(M): 0.178
	(F): 20 (43.5%)	(F): 7 (21.9%)	(F): 0.041 *
Carbonated beverages or prepackaged juices consumed, ≥1 serving/day	(M): 8 (17.4%)	(M): 0 (0%)	(M): 0.094
	(F): 9 (19.6%)	(F): 4 (12.5%)	(F): 0.410
Honey, molasses or jam consumed, ≥1 serving/day or more	(M): 15 (33.3%)	(M): 3 (42.9%)	(M): 0.622
	(F): 7 (15.2%)	(F): 5 (22.7%)	(F): 0.447
Fish or seafood consumed, ≥1 servings/week	(M): 3 (6.4%)	(M): 0 (0%)	(M): 0.332
	(F): 9 (19.6%)	(F): 4 (12.5%)	(F): 0.410
Fat and oils used: vegetable oils (only) ^†^	59 (52.7%)	78 (70.9%)	0.009 *
Olive oils used in dressing and cooking (from total vegetable oil used)	38 (33.9%)	68 (61.8%)	0.002 *
Kind of meat consumed			
Beef	111 (99.1%)	106 (96.4%)	0.56
Lamb	53 (47.3%)	46 (41.8%)	0.97
Beef, chicken and lamb interchangeably	112 (100%)	107 (97.3%)	0.51

Results in the table are related to the percent of cases and controls consuming >3000 kcal/day for men and >2000 kcal/day for women according to the high/low consumption of different food/day/week. Statistical test used: χ^2^-test. * Statistical significance, *p* < 0.05. ^†^ Vegetable oil, such as corn or sunflower oil, are mainly used for cooking, while olive oil is mainly used for dressing. One serving of meat = 120 g; one serving of fish/seafood = 90 g; 1serving of raw vegetables = 1 cup or ½ cup for cooked vegetables; one serving of fruits = 1 fruit or ½ cup of fruit juice; one serving of honey or jam = 1 tablespoon; one serving of dairy products = 1 cup of milk or 40 g of cheese. Abbreviations: NAFLD, non-alcoholic fatty liver disease; F, female; M, male. The cut-off ≥5–6 servings of red meat/week or more was determined according to the study done by Mirmirian et al. on meat consumption between NAFLD participants and controls [34]. The cut-off for raw or cooked vegetables/day, fruits, dairy products, fish or seafood, and honey or molasses was determined according to the Mediterranean Diet Foundation, 2011 [35]. The cut-off for carbonated beverages consumed/day was determined according to the study done by Maersk et al. [36].

## Supplementary Materials

The following are available online at www.mdpi.com/2072-6643/9/11/1245/s1, Figure S1: Results of the Bland Altman analyses (% of energy from protein), Figure S2: Results of the Bland Altman analyses (% of energy from fat), Figure S3: Results of the Bland Altman analyses (% of energy from carbohydrates), Table S1, Food Group.

## Abbreviations

BMI	Body mass index
CDC	Center for Disease Control
CVD	Cardiovascular diseases
DNL	De novo lipogenesis
HDL	High-density lipoprotein
HOMA-IR	Homeostasis model assessment of insulin resistance
IR	Insulin resistance
LDL	Low-density lipoprotein
M/F	Male/female
NAFLD	Nonalcoholic fatty liver disease
NASH	Nonalcoholic steatohepatitis
NF	Natural fructose
OR	Odds ratio
SD	Standard deviation
USDA	United States Department of Agriculture
VLDL	Very-low-density lipoprotein
WHO	World health organization
DRI	Daily recommended intake
EPA	Eicosapentaenoic acid
DHA	Docosahexaenoic acid

## References

[1] Brunt E.M. (2001). Nonalcoholic steatohepatitis: Definition and pathology. Semin. Liver Dis..

[2] Marchesini G., Brizi M., Morselli-Labate A.M., Bianchi G., Bugianesi E., McCullough A.J., Forlani G., Melchionda N. (1999). Association of nonalcoholic fatty liver disease with insulin resistance. Am. J. Med..

[3] Marchesini G., Brizi M., Bianchi G., Tomassetti S., Bugianesi E., Lenzi M., McCullough A.J., Natale S., Forlani G., Melchionda N. (2001). Nonalcoholic fatty liver disease: A feature of the metabolic syndrome. Diabetes.

[4] Abenavoli L., Milic N., Di Renzo L., Preveden T., Medić-Stojanoska M., De Lorenzo A. (2016). Metabolic Aspects of Adult Patients with Nonalcoholic Fatty Liver Disease. World J. Gastroenterol..

[5] Vos M.B., Colvin R., Belt P., Molleston J.P., Murray K.F., Rosenthal P., Schwimmer J.B., Tonascia J., Unalp A., Lavine J.E. (2012). Correlation of Vitamin E, Uric Acid and Diet Composition with Histologic Features of Pediatric Nonalcoholic Fatty Liver Disease. J. Pediatr. Gastroenterol. Nutr..

[6] Zivkovic A.M., German J.B., Sanyal A.J. (2007). Comparative review of diets for the metabolic syndrome: Implications for nonalcoholic fatty liver disease. Am. J. Clin. Nutr..

[7] Toshimitsu K., Matsuura B., Ohkubo I., Niiya T., Furukawa S., Hiasa Y., Kawamura M., Ebihara K., Onji M. (2007). Dietary habits and nutrient intake in non-alcoholic steatohepatitis. Nutrition.

[8] Barclay A.W., Petocz P., McMillan-Price J., Flood V.M., Prvan T., Mitchell P., Brand-Miller J.C. (2008). Glycemic index, glycemic load, and chronic disease risk—A meta-analysis of observational studies. Am. J. Clin. Nutr..

[9] Williams C.M. (2001). Beneficial Nutritional Properties of Olive Oil: Implications for Postprandial Lipoproteins and Factor VII. Nutr. Metab. Cardiovasc. Dis..

[10] Spadaro L.O., Magliocco D., Spampinato S., Piro C., Oliveri C., Alagona C., Papa G., Rabuazzo A.M., Purrello F. (2008). Effects of *n*-3 polyunsaturated fatty acids in subjects with nonalcoholic fatty liver disease. Digest. Liver Dis..

[11] Li Y.H., Yang L.H., Sha K.H., Liu T.G., Zhang L.G., Liu X.X. (2015). Efficacy of Poly-Unsaturated Fatty Acid Therapy on Patients with Nonalcoholic Steatohepatitis. World J. Gastroenterol..

[12] Lambert J.C., Zhou Z., Wang L., Song Z., McClain C.J., Kang Y.J. (2003). Prevention of alterations in intestinal permeability is involved in zinc inhibition of acute ethanol-induced liver damage in mice. J. Pharmacol. Exp. Ther..

[13] Koek G.H., Liedorp P.R., Bast A. (2011). The role of oxidative stress in non-alcoholic steatohepatitis. Clin. Chim. Acta.

[14] Lustig R.H. (2010). Fructose: Metabolic, Hedonic, and Societal Parallels with Ethanol. J. Am. Diet. Assoc..

[15] Abdelmalek M.F., Suzuki A., Guy C., Unalp-Arida A., Colvin R., Johnson R.J., Diehl A.M. (2010). Nonalcoholic Steatohepatitis Clinical Research Network: Increased fructose consumption is associated with fibrosis severity in patients with nonalcoholic fatty liver disease. Hepatology.

[16] Oddy W., Herbison C., Jacoby P., Adams L. (2013). The Western Dietary Pattern Is Prospectivelya associated with Nonalcoholic Fatty Liver Disease in Adolescence. Am. J. Gastroenterol..

[17] Ryan M.C., Itsiopoulos C., Thodis T., Ward G., Trost N., Hofferberth S., O’Dea K., Desmond P.V., Johnson N.A., Wilson A.M. (2013). The Mediterranean Diet Improves Hepatic Steatosis and Insulin Sensitivity in Individuals with Non-Alcoholic Fatty Liver Disease. J. Hepatol..

[18] Koch M., Nöthlings U., Lieb W. (2015). Dietary Patterns and Fatty Liver Disease. Curr. Opin. Lipidol..

[19] Landrivon G., Delahaye F. (1995). La Recherche Clinique De l’Idée à la Publication.

[20] Zelber-Sagi S., Nitzan-Kaluski D., Goldsmith R., Webb M., Blendis L., Halpern Z., Oren R. (2007). Long term nutritional intake and the risk for non-alcoholic fatty liver disease (NAFLD): A population based study. J. Hepatol..

[21] Yu E., Rimm E., Qi L., Rexrode K., Albert M.A., Sun Q., Willett W.C., Hu F.B., Manson J.E. (2016). Diet, Lifestyle, Biomarkers, Genetic Factors, and Risk of Cardiovascular Disease in the Nurses Health Studies. Am. J. Public Health.

[22] Cade J., Thompson R., Burtley V., Warm D. (2002). Development, validation and utilization of food frequency questionnaire—A review. Public Health Nutr..

[23] Alberti K.G., Eckel R.H., Grundy S.M., Zimmet P.Z., Cleeman J.I., Donato K.A., Fruchart J.-C., James W.P., Loria C.M., Smith S.C. (2009). Harmonizing the metabolic syndrome: A joint interim statement of the International Diabetes Federation Task Force on Epidemiology and Prevention; National Heart, Lung, and Blood Institute; American Heart Association; World Heart Federation; International Atherosclerosis Society; and International Association for the Study of Obesity. Circulation.

[24] Muniyappa R., Lee S., Chen H., Quon M.J. (2008). Current approaches for assessing insulin sensitivity and resistance in vivo: Advantages, limitations, and appropriate usage. Am. J. Physiol. Endocrinol. Metab..

[25] Stern S.E., Williams K., Ferrannini E., DeFronzo R.A., Bogardus C., Stern M.P. (2005). Identification of individuals with insulin resistance using routine clinical measurements. Diabetes.

[26] Papazian T., Hout H., Sibai D., Helou N., Younes H., El Osta N., Khabbaz L.R. (2016). Development, Reproducibility and Validity of a Food Frequency Questionnaire among Pregnant Women Adherent to the Mediterranean Dietary Pattern. Clin. Nutr..

[27] Nelson M., Atkinson M., Darbyshire S. (1996). Food photography II: Use of food photographs for estimating portion size and the nutrient content of meals. Br. J. Nutr..

[28] The Nutrient Data Laboratory Food Composition Table (FCT), Food and Nutrition Information Center, United State Department of Agriculture (USDA). https://ndb.nal.usda.gov/ndb/.

[29] Distefano C., Zhu M., Mindrilla D. (2009). Understanding and using factor scores: Considerations for the applied researcher. Pract. Assess. Res. Eval..

[30] Waked M., Salameh P., Aoun Z. (2009). Water-pipe (narguile) smokers in Lebanon: A pilot study. East. Mediterr. Health J..

[31] Ainsworth B.E., Haskell W.L., Leon A.S., Jacobs D.R., Montoye H.J., Sallis J.F., Paffenbarger R.S. (1993). Compendium of physical activities: Classification of energy costs of human physical activities. Med. Sci. Sports Exerc..

[32] (2004). Guidelines for Data Processing and Analysis of the International Physical Activity Questionnaire (IPAQ)-Short Form, Version 2.0. http://www.ipaq.ki.se.

[33] Adjustment for Total Energy Intake in Epidemiologic Studies. http://ajcn.nutrition.org/content/65/4/1220S.abstract.

[34] Mirmiran P., Amirhamidi Z., Ejtahed H.S., Bahadoran Z., Azizi F. (2017). Relationship between Diet and Non-alcoholic Fatty Liver Disease: A Review Article. Iran. J. Public Health.

[35] Davis C., Bryan J., Hodgson J., Murphy K. (2015). Definition of the Mediterranean Diet: A Literature Review. Nutrients.

[36] Maersk M., Belza A., Stødkilde-Jørgensen H., Ringgaard S., Chabanova E., Thomsen H., Pedersen S.B., Astrup A., Richelsen B. (2012). Sucrose-sweetened beverages increase fat storage in the liver, muscle, and visceral fat depot: A 6-mo randomized intervention study. Am. J. Clin. Nutr..

[37] Marchesini G., Bugianesi E., Forlani G., Cerrelli F., Lenzi M., Manini R., Natale S., Vanni E., Villanova N., Melchionda N. (2003). Nonalcoholic fatty liver, steatohepatitis, and the metabolic syndrome. Hepatology.

[38] Dowman J.K., Tomlinson J.W., Newsome P.N. (2010). Pathogenesis of non-alcoholic fatty liver disease. QJM.

[39] Illouz F., Roulier V., Rod A., Gallois Y., Pellé C.-P., Aubé C., Rohmer V., Ritz P., Ducluzeau P.H. (2008). Distribution of adipose tissue: Quantification and relationship with hepatic steatosis and vascular profiles of type 2 diabetic patients with metabolic syndrome. Diabetes Metab..

[40] Ghamar-Chehreh M.E., Khedmat H., Amini M., Taheri S. (2013). Predictive value of having positive family history of cardiovascular disorders, diabetes mellitus, dyslipidemia, and hypertension in non-alcoholic fatty liver disease patients. Acta Med. Iran..

[41] Krauss R.M. (2004). Lipids and Lipoproteins in Patients with Type 2 Diabetes. Diabetes Care.

[42] Zelber-Sagi S., Ratziu V., Oren R. (2011). Nutrition and physical activity in NAFLD: An overview of the epidemiological evidence. World J. Gastroenterol..

[43] Church T.S., Kuk J.L., Ross R., Priest E.L., Biltoff E., Blair S.N. (2006). Association of Cardio Respiratory Fitness, Body Mass Index, and Waist Circumference to Nonalcoholic Fatty Liver Disease. Gastroenterology.

[44] Hannukainen J.C., Nuutila P., Borra R., Ronald B., Kaprio J., Kujala U.M., Janatuinen T., Heinonen O.J., Kapanen J., Viljanen T. (2007). Increased physical activity decreases hepatic free fatty acid uptake: A study in human monozygotic twins. J. Physiol. (Lond.).

[45] Tamura Y., Tanaka Y., Sato F., Choi J.B., Watada H., Niwa M., Kinoshita J., Ooka A., Kumashiro N., Igarashi Y. (2005). Effects of diet and exercise on muscle and liver intracellular lipid contents and insulin sensitivity in type 2 diabetic patients. J. Clin. Endocrinol. Metab..

[46] Naja F., Hwalla N., Itani L., Salem M., Azar S.T., Zeidan M.N., Nasreddine L. (2012). Dietary patterns and odds of Type 2 diabetes in Beirut, Lebanon: A case–control study. Nutr. Metab..

[47] Osler M., Helms Andreasen A., Heitmann B., Høidrup S., Gerdes U., Mørch Jørgensen L., Schroll M. (2002). Food intake patterns and risk of coronary heart disease: A prospective cohort study examining the use of traditional scoring techniques. Eur. J. Clin. Nutr..

[48] Kontogianni M.D., Tileli N., Aikaterini M., Georgoulis M., Deutsch M., Tiniakos D., Fragopoulou E., Zafiropoulou R., Manios Y., Papatheodoridis G. (2014). Adherence to the Mediterranean Diet Is Associated with the Severity of Non-Alcoholic Fatty Liver Disease. Clin. Nutr..

[49] Kouris-Blazos A., Gnardellis C., Wahlqvist M.L., Trichopoulos D., Lukito W., Trichopoulou A. (1999). Are the advantages of the Mediterranean diet transferable to other populations? A cohort study in Melbourne, Australia. Br. J. Nutr..

[50] Trichopoulou A., Costacou T., Bamia C., Trichopoulos D. (2003). Adherence to a Mediterranean Diet and Survival in a Greek Population. N. Engl. J. Med..

[51] MacQueen H.A., Sadler D.A., Moore S.A., Daya S., Brown J.Y., David E., Shuker D.E. (2007). Deleterious effects of a cafeteria diet on the livers of nonobese rats. Nutr. Res..

[52] Schulze M.B., Manson J.E., Ludwig D.S., Colditz G.A., Stampfer M.J., Willett W.C., Hu F.B. (2004). Sugar-Sweetened Beverages, Weight Gain, and Incidence of Type 2 Diabetes in Young and Middle-Aged Women. JAMA.

[53] Nasreddine L., Hwalla N., Sibai A., Hamzé M., Parent-Massin D. (2006). Food consumption patterns in an adult urban population in Beirut, Lebanon. Public Health Nutr..

[54] Schwenzer N.F., Springer F., Schraml C., Stefan N., Machann J., Schick F. (2009). Non-Invasive Assessment and Quantification of Liver Steatosis by Ultrasound, Computed Tomography and Magnetic Resonance. J. Hepatol..

[55] Kani A.H., Alavian S.M., Esmaillzadeh A., Adibi P., Azadbakht L. (2013). Dietary Quality Indices and Biochemical Parameters among Patients with Non Alcoholic Fatty Liver Disease (NAFLD). Hepat. Mon..

